# Habitat Suitability and Driving Factors of *Cycas panzhihuaensis* in the Hengduan Mountains

**DOI:** 10.3390/plants14172797

**Published:** 2025-09-06

**Authors:** Yuting Ding, Yuanfeng Yang, Xuefeng Peng, Juan Wang, Mengjie Wu, Ying Zhang, Xing Liu, Peihao Peng

**Affiliations:** 1 College of Geography and Planning, Chengdu University of Technology, Chengdu 610059, China; 2023010044@stu.cdut.edu.cn (Y.D.); yuanfengyang125@gmail.com (Y.Y.); idwangjuan@cdut.edu.cn (J.W.); mengjiewu0604@163.com (M.W.); 529213949@139.com (Y.Z.); llxxxlxx@163.com (X.L.); 2 Institute of Mountain Hazards and Environment, Chinese Academy of Sciences, Chengdu 610213, China; piceapen@163.com; 3 University of Chinese Academy of Sciences, Beijing 100049, China

**Keywords:** *Cycas panzhihuaensis*, habitat suitability, environmental drivers, conservation prioritization, Geodetector, Hengduan Mountains

## Abstract

The Hengduan Mountains, a global biodiversity hotspot, harbor numerous endemic plant species shaped by complex topography and microclimatic variation. However, increasing habitat fragmentation due to human activities threatens narrowly distributed species such as *Cycas panzhihuaensis*. To investigate its habitat suitability and inform conservation, we applied the MaxEnt model, Geodetector, and Zonation to predict potential distribution, identify key environmental drivers, and delineate priority conservation areas. Our results show that only 18.36% of the region constitutes suitable and highly fragmented habitat, primarily concentrated along the dry–hot valleys of the Jinsha and Yalong Rivers, and it is shrinking while shifting southward and southeastward under climate change. Elevation emerged as the dominant driver (*q* = 0.45), with strong interaction effects among topographic, climatic, soil, and anthropogenic factors, highlighting the role of environmental synergies in shaping habitat heterogeneity. Priority conservation areas covered 32% of suitable habitat and overlapped only 6.17% with existing protected areas, indicating a spatial conservation gap. These findings emphasize the need to incorporate microhabitat heterogeneity and environmental interactions in conservation planning and support the adoption of micro-reserve strategies to complement existing reserves. Our study provides a practical framework for protecting vulnerable montane species and offers insights into plant distribution dynamics in topographically complex regions.

## 1. Introduction

The Hengduan Mountains region (HDM) in southwestern China is not only recognized as one of the Earth’s 36 biodiversity hotspots [1] but also serves as a critical Quaternary glacial refugium for relict plant species in East Asia [2], harboring unique and rich biodiversity. Yet, over the past three decades, rapid infrastructure development and ongoing agricultural expansion have exerted unprecedented pressure, resulting in significant declines in natural vegetation cover and intensified habitat fragmentation [3,4,5]. Amid these challenges, current conservation efforts face two major constraints: (1) the existing protected area network covers only 17.9% of the HDM, reflecting limited spatial coverage and severely inadequate connectivity among protected areas [6]; and (2) species distribution modeling (SDM) and conservation planning have predominantly focused on climatic variables [7,8]. However, it is often overlooked that topographic factors play a critical regulatory role by buffering extreme climatic events and creating heterogeneous microhabitats, especially in microrefugia such as dry–hot valleys.

In this context, conducting in-depth studies on flagship species that are highly sensitive to habitat changes and indicative of key regional ecological processes is of great significance. *Cycas panzhihuaensis*, a relict species with high evolutionary significance within the Cycadaceae [9,10], is also a representative lithophyte in the dry–hot valley ecosystems of the HDM [11]. Ecologically, as one of the dominant woody plants in the dry–hot valley shrub communities, *C. panzhihuaensis* contributes to slope stabilization, soil–water conservation, and the maintenance of microhabitat heterogeneity, thereby playing an irreplaceable role in sustaining ecosystem functions and biodiversity in these fragile montane river valleys [12]. Despite its ecological importance, this species has long been subjected to multiple survival threats. Distributed in the dry–hot valleys of the Jinsha River and its tributaries, spanning southern Sichuan and northern Yunnan, *C. panzhihuaensis* has experienced dramatic population declines at low elevations due to historical overharvesting [13], while anthropogenic disturbances have caused severe habitat fragmentation. Moreover, *C. panzhihuaensis* predominantly inhabits steep slopes composed of dry limestone soils, shale, and sandstone substrates. The harsh native conditions contribute to its low regeneration rates [14]. In 2022, the species was classified as vulnerable by the International Union for Conservation of Nature (IUCN) [15]. Nevertheless, research on *C. panzhihuaensis* has mainly focused on physiological responses [16,17], population genetics [14,18], and phylogeography [14,18], while studies evaluating habitat suitability and the relative influence of environmental and anthropogenic factors are still limited. Given that the distribution pattern of *C. panzhihuaensis* is jointly shaped by climate, topography, soil, and human activities, it provides an ideal natural model for investigating the interactive effects of environmental variables and the underlying mechanisms driving species distribution patterns.

Recently, SDMs have gained wide recognition in the conservation of endangered plants. Among these, the Maximum Entropy (MaxEnt) model is widely employed for predicting potential suitable habitats and informing conservation strategies for endangered flora [19,20] and fauna [21,22], owing to its robust performance with small sample sizes. This approach provides a critical theoretical foundation for evidence-based conservation planning. Complementarily, as a spatial statistical tool, Geodetector offers the capability to quantify the independent and interactive explanatory power of diverse environmental factors on species distributions [23], thereby significantly complementing SDMs by enhancing the mechanistic understanding of underlying drivers. Moreover, identification tools for priority conservation areas are needed to transform the prediction results of suitable habitats into spatial conservation strategies. The Zonation software (version 5), recognized for its strengths in integrating multi-source spatial data (e.g., species occurrence, connectivity, existing protected areas) and optimizing conservation efficiency, has been extensively applied in terrestrial and marine ecosystems [24,25].

In summary, this study focuses on *C. panzhihuaensis* as the research subject, integrating the MaxEnt model, Geodetector, and Zonation conservation optimization methods. Based on multidimensional environmental variables including climate, topography, soil, and human activities, the objectives are to: (1) identify the distribution patterns of potential suitable habitats; (2) quantitatively analyze the independent and interactive driving mechanisms of climatic and non-climatic factors on its distribution; and (3) delineate priority conservation areas and evaluate the effectiveness of the existing protected area network. This research contributes to a deeper understanding of the ecological mechanisms of species endemic to dry–hot valleys, provides scientific support for the precise conservation of *C. panzhihuaensis*, and offers a theoretical foundation and applied framework for habitat suitability assessment and conservation planning of rare plants in the HDM.

## 2. Results

### 2.1. MaxEnt Performance and Environmental Contributions

The ROC curves (Figure S1) generated by the MaxEnt model indicated a mean AUC value of 0.945 (±0.042) across ten replicate runs, suggesting strong predictive performance with no signs of overfitting [26]. Overall, the model was stable and reliable.

As shown in Table 1, the Jackknife test for variable contributions and permutation importance identified six environmental variables with individual contributions exceeding 5%, collectively accounting for 88.4% of the total. Among them, elevation was the most influential factor, contributing 43.1%, significantly higher than other variables, indicating that it plays a dominant role in shaping the vertical distribution pattern of *C. panzhihuaensis*. Among climatic variables, mean diurnal range (bio2), precipitation of driest month (bio14), and precipitation of warmest quarter (bio18) jointly contributed more than 29%, reflecting the species’ sensitivity to both precipitation and temperature variability. Although the Human Footprint Index (HFI) contributed only 8.8%, it represents an exogenous disturbance that may constrain the distribution at habitat margins. In contrast, soil variables (exchangeable calcium and pH) showed relatively low importance, with a combined contribution of 8.5%.

**Figure 1:**
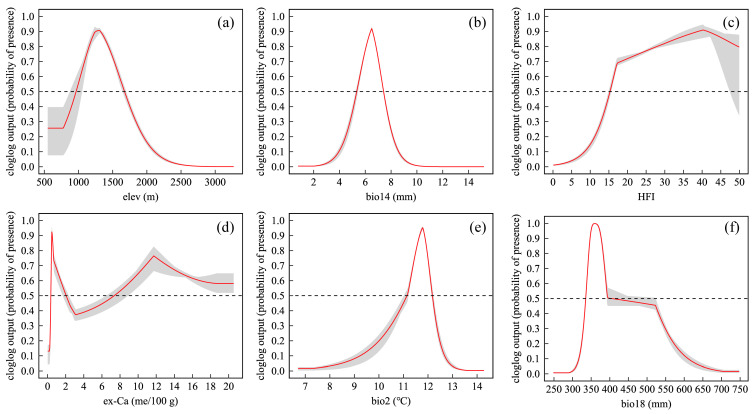
Response curves of six key variables affecting the predicted distribution probability of *Cycas panzhihuaensis*. (**a**) elev; (**b**) bio14; (**c**) HFI; (**d**) ex-Ca; (**e**) bio2; (**f**) bio18 The red lines indicate the predicted probability of presence, the gray shaded areas represent the 95% confidence intervals, and the black dashed lines mark the 0.5 suitability threshold. Refer to Table 1 for the meaning of the bioclimatic variables.

The response curves further elucidated the ecological preferences of *C. panzhihuaensis*, as shown in Figure 1, by illustrating the relationship between individual environmental variables and the species’ probability of presence [26]. Suitable ranges of six key variables were identified where the predicted presence exceeded 0.5 [27]. *C. panzhihuaensis* occurred most frequently at elevations of 966–1673 m, with bio14 values of 5–7 mm, HFI between 15 and 50, ex-Ca from 7 to 19 me/100 g, bio2 concentrations of 11–12 °C, and bio18 ranging from 336 to 405 mm.

### 2.2. Spatial Patterns of Suitable Habitats for C. panzhihuaensis

#### 2.2.1. Current Potential Distribution

**Figure 2:**
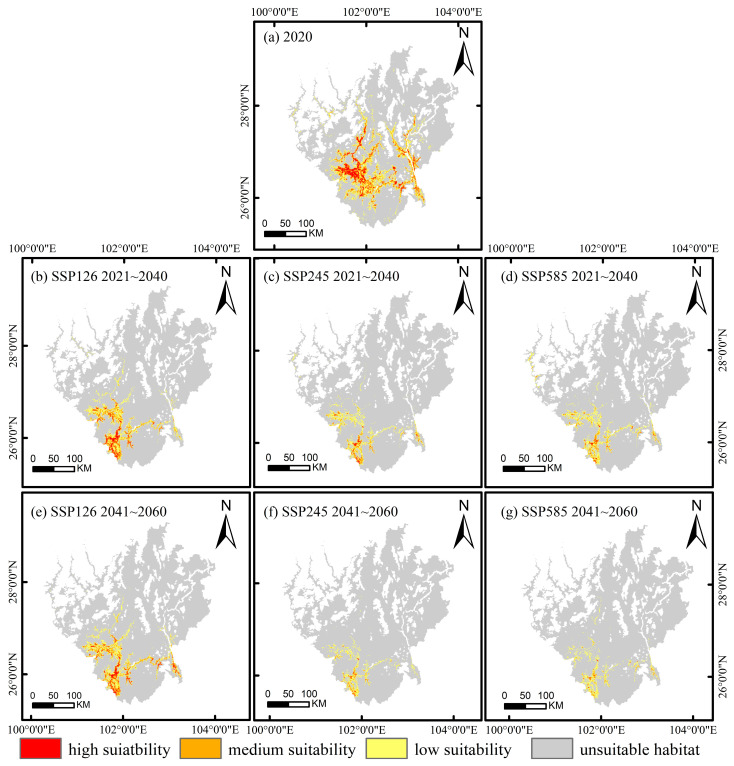
Spatial distribution of suitable habitats for *C. panzhihuaensis* in the current period and under future climate scenarios.

The MaxEnt model predicted a total suitable area of 12,541.77 km^2^ for *C. panzhihuaensis*, accounting for 18.36% of the study area. Figure 2a shows that these suitable habitats are mainly distributed in a belt-like pattern along the main streams of the Jinsha and Yalong Rivers, forming a fragmented spatial configuration that suggests potential threats to habitat integrity. Among them, high, medium, low, and unsuitable habitat suitability cover 2056.92 km^2^ (3.01%), 3549.03 km^2^ (5.19%), and 6935.82 km^2^ (10.16%) of the study area, respectively. The remaining 81.64% (55,780.19 km^2^) is classified as unsuitable habitat.

#### 2.2.2. Future Potential Distribution Under Climate Change

Under different climate scenarios, the future suitable habitat range of this species exhibits a significant contraction trend (Table 2), accompanied by a spatial shift towards the south and southeast (Figure 2b–g). Under the SSP126 low emission scenario, the suitable area is projected to decrease by 53.52% to 5830.13 km^2^ during 2021–2040, followed by a slight recovery to 6515.75 km^2^ in 2041–2060. Some originally unsuitable areas are expected to transition into low and medium suitability categories, indicating a certain potential for natural recovery under this scenario. Under the SSP245 medium emission scenario, the suitable area continues to decline, reaching only 2276.27 km^2^ by 2041–2060, with the highly suitable area sharply decreasing to 68.02 km^2^. Although the total suitable area under the SSP585 high emission scenario is slightly larger than that under SSP245, the expansion consists primarily of low suitability areas, reflecting notable habitat quality degradation that may adversely affect the long term survival and healthy development of the population.

Spatial pattern analysis reveals that suitable habitats are relatively concentrated in counties such as Yongren, Yuanmou, Huaping, Renhe, Wuding and Huili, whereas in counties including Miyi, Yanbian, Yanyuan, Dechang, Ningnan, Qiaojia, Dongchuan, Huidong and Luquan, the distribution is fragmented and the suitability remains relatively stable. In conclusion, the suitable habitat of *C. panzhihuaensis* faces severe contraction under future climate conditions, highlighting the need for enhanced monitoring and conservation efforts to mitigate the ecological risks posed by climate change.

### 2.3. Identification of Driving Factors

**Figure 3:**
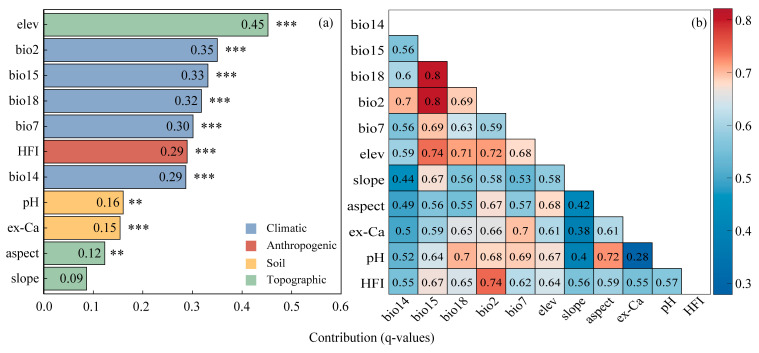
Geodetector-based analysis of environmental influences on the distribution of *C. panzhihuaensis*: (**a**) explanatory power (*q*-values) of individual environmental factors; (**b**) interaction effects between paired environmental factors. Significance levels: *** *p* < 0.001; ** *p* < 0.01.

The factor detector was applied to assess the explanatory power of each environmental variable on the potential distribution of *C. panzhihuaensis*. All factors except for slope were statistically significant (*p* < 0.01), indicating their strong associations with the species distribution (Figure 3a). Among them, elevation (*q* = 0.45) emerged as the dominant factor, indicating its primary role in shaping the suitable habitat. All climatic factors and the human footprint index (HFI) were categorized as important factors, with *q*-values exceeding 0.29, which highlights the significant role of climatic conditions and anthropogenic pressure in shaping the distribution pattern of *C. panzhihuaensis*. All soil factors and aspect showed moderate explanatory power (0.12 < *q* < 0.20), which were considered secondary factors. In contrast, slope (*q* = 0.09) exhibited the weakest explanatory power and was not statistically significant, possibly due to the species’ broad tolerance to micro-topographic variation within its native range.

The interaction detection results (Figure 3b) revealed that all 11 environmental factors exhibited enhanced interactive effects, exceeding the explanatory power of single factors. Notably, the interactions between precipitation seasonality (bio15) and warmest quarter precipitation (bio18), as well as mean diurnal temperature range (bio2), yielded the highest *q*-values (0.80), surpassing the sum of their respective individual *q*-values (0.65 and 0.68). This indicated a strong synergistic regulation among climatic factors. These findings suggested that the distribution pattern of *C. panzhihuaensis* was not driven by any single factor, but rather results from complex coupling mechanisms among multiple climatic, topographic, soil, and anthropogenic drivers.

### 2.4. Determination of Conservation Prioritization

**Figure 4:**
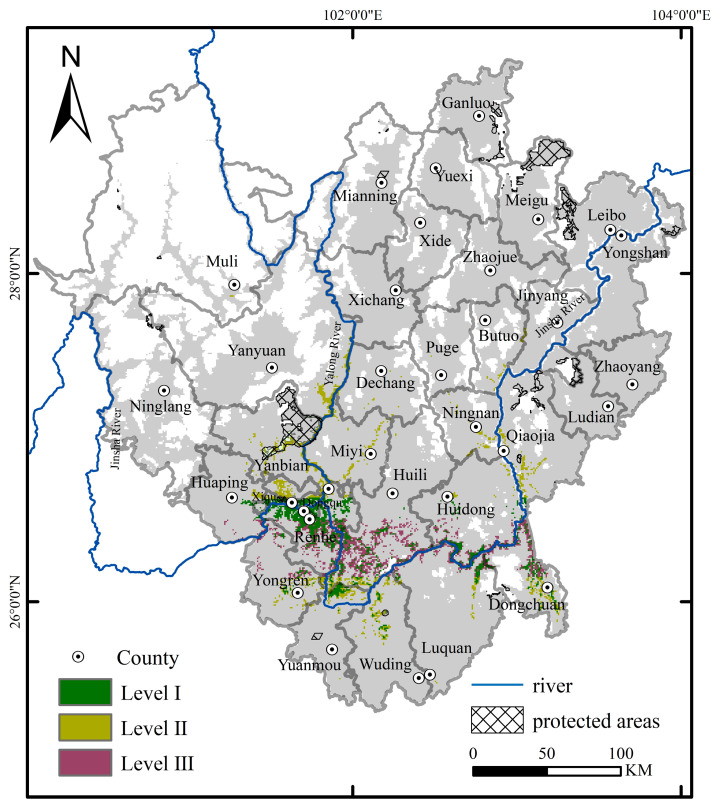
Spatial distribution of priority conservation areas for *C. panzhihuaensis* based on Zonation analysis.

A map of priority conservation areas for *C. panzhihuaensis* was generated by Zonation 5 (Figure 4), illustrating the spatial distribution characteristics with varying conservation prioritization. Overall, the priority conservation areas covered 3671.90 km^2^, accounting for 29.30% of the suitable habitat. They were primarily concentrated in the mid-to high-elevation zones of the southern and western parts of the study region (such as Renhe, Xiqu, Dongqu, Huili), particularly in the mountainous river valleys near the confluence of the Jinsha and Yalong Rivers. This distribution closely aligned with the core occurrence areas identified through field surveys and corresponded well with the species’ actual ecological requirements.

The spatial coverage efficiency of the current conservation system was assessed by overlaying the distribution of existing protected areas, as shown in Table 3. The results showed that Level I areas total 1246.77 km^2^, of which only 0.71% were covered by existing protected areas. Level II areas had 1200.32 km^2^, with 5.46% falling within current protected areas, indicating relatively higher coverage. Notably, Level III areas, covering 1227.81 km^2^, were entirely unrepresented within the existing protected area network.

## 3. Discussion

### 3.1. Fragmented Habitat and Conservation Challenges of C. panzhihuaensis

The MaxEnt model revealed that the potential suitable habitats of *C. panzhihuaensis* are characterized by a highly restricted, belt-like distribution along the dry–hot valleys of the Jinsha River at the Sichuan–Yunnan border. This prediction aligns well with both historical records and field investigations [11,13], highlighting the species’ close association with the deep, sheltered river valley microclimate, where high temperatures, scarce rainfall, and rugged topography create a unique ecological niche. The steep altitudinal gradients, arid climate, and highly seasonal precipitation of the Jinsha River valley together create a highly heterogeneous microenvironment [28], which fundamentally drives the fragmentation of suitable habitats. This extremely narrow and fragmented distribution pattern stands in sharp contrast to the broad distribution of other *Cycas* species [29,30], and suggests that *C. panzhihuaensis* has evolved an extremely narrow ecological niche. This niche specialization is likely rooted in its history as a glacial refugial relict and its adaptive specialization to hot and dry stress conditions [31].

On top of its inherent ecological fragility, topographic barriers and limited water resources severely restrict spatial connectivity among populations. Field investigations in 1999 documented 13 natural populations of *C. panzhihuaensis,* mainly distributed in the dry–hot valleys of the Jinsha River and its tributaries [13], most of which are now significantly isolated. Since 1979, commercial harvesting has resulted in the extinction of eight populations, causing a dramatic reduction in its range from 30,700 km^2^ to just 8800 km^2^, with remaining habitat fragments highly localized and severely fragmented [12]. Meanwhile, human activities such as infrastructure development and mining have further encroached upon and subdivided the remaining habitat patches, exacerbating habitat fragmentation and further reducing landscape connectivity [14]. This fragmented landscape has brought about notable ecological consequences: numerous small populations are confined to limited habitat patches and face the dual threats of genetic drift and inbreeding depression. Simultaneously, reduced gene flow intensifies isolation effects, leading to declines in genetic diversity and adaptive potential [32].

Under climate change, the suitable habitat of *C. panzhihuaensis* has shown a trend of shifting southward and southeastward. Most of these newly formed suitable areas are classified as low suitability, demonstrating a characteristic pattern of areal expansion accompanied by quality degradation. Currently, fragmented yet relatively stable habitat patches remain in counties such as Miyi and Yanbian, which can function as important refugia for this species. Therefore, it is recommended that conservation priorities focus on protecting existing stable populations and the ecological corridors facilitating their southward migration, so as to enhance the species’ capacity for ongoing adaptation to climate change.

### 3.2. Dominance of Elevation and Multi-Factor Synergy

Further exploration of the driving factors revealed that elevation is the primary determinant of the spatial distribution of *C. panzhihuaensis* (Figure 3a). In the HDM, characterized by dramatic topographic relief and complex environmental gradients, the explanatory power of elevation significantly exceeds that of other environmental factors. This finding challenges the conventional view that climate is the dominant driver of large-scale distribution patterns [33], and highlights that in mountainous terrains, elevation acts as a proxy for climate, topography, soil, and human activities, thereby exerting a more direct and comprehensive influence on species distribution.

It is worth noting that in the interaction detection analysis, the synergistic effects between elevation and climatic factors were particularly pronounced (Figure 3b). Specifically, the interactions between elevation and bio15, bio2, and bio18 yielded *q*-values exceeding 0.71, higher than those of the single factors. This elevation-climate synergy illustrates the regulatory role of topography on regional climatic gradients and reveals the complex ecological responses of *C. panzhihuaensis* to multiple environmental drivers. The pronounced response of *C. panzhihuaensis* to climatic variables can be attributed to its distinct ecological and physiological adaptations to dry–hot valley environments. Precipitation seasonality (bio15), reflecting the marked alternation between wet and dry seasons, exerts a decisive influence on water availability. This factor is particularly critical for cycads with shallow root systems and inherently low regeneration rates, as it directly regulates seedling establishment and adult water-use efficiency [34]. Precipitation of the warmest quarter (bio18) further determines drought stress intensity during the hottest period of the year; insufficient rainfall at this stage substantially constrains survival and recruitment, especially on exposed slopes with shallow soils [34]. In parallel, mean diurnal temperature range (bio2) affects photosynthetic activity and carbon balance under pronounced day–night thermal contrasts. Large diurnal fluctuations increase night-time respiratory costs while potentially limiting daytime photosynthesis, thereby reducing net carbon gain and constraining growth in microsites exposed to extreme thermal variation [35]. Collectively, these results indicate that the distribution of *C. panzhihuaensis* is strongly constrained by climatic variables associated with both water availability and temperature variability, which jointly shape its narrow ecological niche.

Similar patterns have been observed in various mountainous ecosystems. For example, studies in the HDM have shown that topographic heterogeneity and paleoclimatic fluctuations jointly shape regional biodiversity and endemism patterns, often surpassing the explanatory power of individual modern climate factors [36]. Additionally, in the Qilian Mountains, the interaction between elevation and annual precipitation significantly influences the spatial pattern of NDVI, reflecting the key role of topography-climate interplay in vegetation distribution [37]. These results suggest that the elevation-climate synergistic mechanisms may be widespread in topographically complex mountainous regions, underscoring the importance of re-evaluating species distribution models within such environmental contexts.

**Figure 5:**
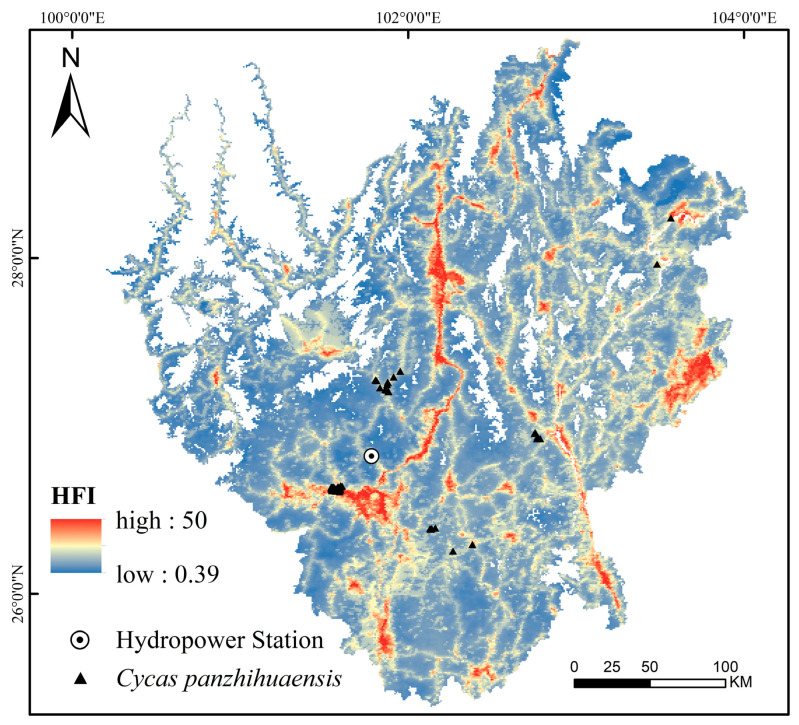
Spatial patterns of HFI in the study area.

Moreover, the interaction between elevation and the Human Footprint Index (HFI) also showed substantial explanatory power (*q* = 0.57), indicating the pivotal role of topography in modulating or amplifying anthropogenic impacts [38]. Specifically, elevation influences human activity intensity indirectly by modulating temperature, humidity, solar radiation, and microhabitat heterogeneity [38]. This is evident in the contrasting patterns across elevation gradients: while low-elevation valley regions possess abundant thermal resources, the combined effects of foehn-induced aridity, sparse vegetation, and intense human activity have resulted in pronounced habitat fragmentation [39]. In contrast, mid-to high-elevation zones, despite being cooler, retain relatively continuous and semi-natural habitat patches that serve as crucial refugia for *C. panzhihuaensis*. Notably, the HFI map (Figure 5) revealed that *C. panzhihuaensis* populations are frequently located in areas with relatively high HFI values, rather than exclusively in low-disturbance refugia. This pattern reflects not only the species’ ecological preferences but also historical and ongoing land-use transformations. For instance, the construction of the Ertan Hydropower Station in the 1990s markedly expanded water bodies and reshaped riparian habitats (Figure S2). Similarly, since the 1960s, large-scale afforestation programs have replaced native shrublands with commercial plantations (Figure S2), thereby altering local microclimates, soil conditions, and competitive dynamics. These human-induced changes have intensified landscape fragmentation, forcing *C. panzhihuaensis* into micro-refugia such as limestone cliffs and steep slopes, where direct disturbance is reduced but surrounding human pressure remains high. Therefore, elevation functions not only as a direct ecological factor but also as a spatial proxy that integrates both natural gradients and anthropogenic stressors, providing critical insight into the niche structure and conservation needs of *C. panzhihuaensis*.

**Figure 6:**
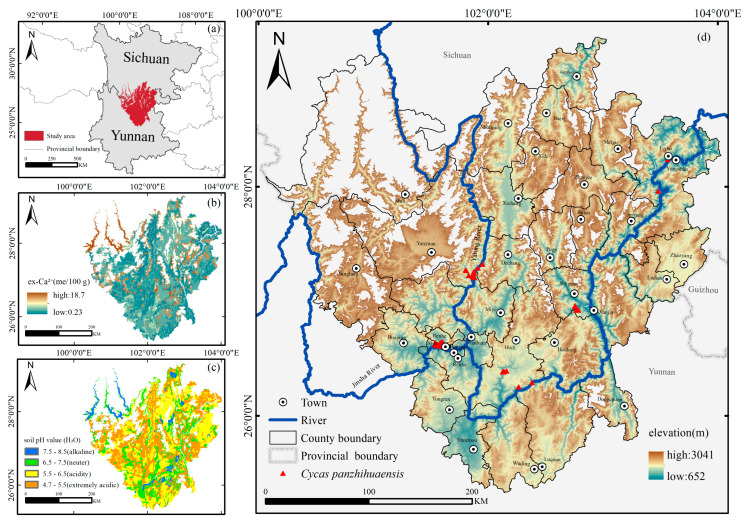
Overview of the study area: (**a**) regional location; (**b**) exchangeable calcium (me/100 g); (**c**) soil pH; (**d**) elevation distribution with occurrence records.

In addition, elevation serves as an integrative indicator of soil properties. *C. panzhihuaensis* predominantly inhabits steep slopes derived from limestone, shale, and sandstone substrates [11,14]. Its pronounced dependence on calcareous bedrock (Figure 6b) and specific soil pH conditions (Figure 6c) highlights a distinctive niche in soil conditions. In low-elevation dry–hot valleys, frequent foehn winds, arid conditions, and intense evapotranspiration accelerate slope erosion, removing the fine, organic-rich topsoil and exposing calcareous substrates, which in turn promotes the development of alkaline, calcium-rich soils [40,41]. Within these areas, slope position further modulates soil properties: foot-slopes and gentler mid-slopes tend to accumulate sediments and nutrients, resulting in relatively higher soil fertility, while upper slopes are more erosion-prone and consequently exhibit lower levels of organic matter and exchangeable cations [42]. Conversely, mid-to high-elevation areas are less affected by foehn winds and are characterized by cooler, moister climates and more stable soil hydrothermal regimes [43], allowing soil pH and exchangeable calcium levels to remain within a moderate range [40]. These conditions are generally more conducive to sustaining suitable habitats for *C. panzhihuaensis*, which is consistent with our field survey observations of the species’ occurrence points.

In summary, our study reveals the synergistic regulation of plant distribution by elevation, climate, human disturbance, and soil. This offers valuable insights for predicting distribution patterns and formulating targeted conservation strategies for endangered species in topographically complex regions. It also underscores the importance of considering the coupled mechanisms among climatic, topographic, soil, and anthropogenic factors in future ecological modeling. Meanwhile, our findings suggest that under the unique geographic setting of the HDM, the complex ecological gradients represented by elevation impose stronger and more direct constraints on the distribution pattern of *C. panzhihuaensis* than any single environmental factor. This understanding provides a region-specific theoretical basis for interpreting the ecological niche structure, adaptive strategies, and conservation planning of rare mountain plants.

### 3.3. Gaps in Exiting Protected Area Network

This study identified priority conservation areas for *C. panzhihuaensis* and assessed their spatial congruence with the existing protected area network. The results revealed that only 6.17% of the identified priority habitats fall within the current protected areas, indicating a substantial spatial mismatch between the established conservation network and the critical habitat requirements of this species. Similar spatial inconsistencies have been reported for other taxa and mountainous regions worldwide [44,45], underscoring a global challenge in aligning species-specific priority conservation with existing protected area configurations.

This spatial misalignment may be attributable to the historical planning process, which often overlooked the microhabitat requirements of rare or range-restricted plants, and the pressing constraints imposed by socioeconomic demands on land use [45,46]. Consequently, many priority habitats remain outside formal protection or lie adjacent to areas of intense human activity, leaving them vulnerable to threats such as land-use change, agricultural expansion, and infrastructure development. These pressures collectively compromise the long-term viability of *C. panzhihuaensis* populations and undermine the broader integrity of regional biodiversity.

Most existing protected areas in the HDM are primarily established for forest ecosystems [33], which limits their capacity to cover the heterogeneous microhabitats required by many rare and endangered plant species. Relying solely on large-scale protected areas presents significant protection gaps and management limitations. For instance, although the wild populations of *C. panzhihuaensis* within the established nature reserves are relatively well protected, in our survey wild individuals outside these protected areas often occur as isolated single or few plants scattered across the landscape. These remnant individuals face heightened risks of local extinction due to their small population sizes, limited reproductive potential, and increased vulnerability to environmental and human disturbances.

To address the gaps in the current protected area network, we propose complementary conservation measures for key counties that host *C. panzhihuaensis* populations currently lying outside existing protected areas (Table 3). These counties represent critical habitats partially overlapping with modeled priority conservation areas but remain insufficiently protected. Given the fragmented distribution and socio-economic constraints, we recommend a flexible, cost-effective approach focused on establishing “micro-reserves” or community-managed conservation units. Such small-scale protected sites can be integrated into existing land-use systems, providing targeted protection for isolated populations while minimizing resource investment [47,48]. Equally important, the long-term effectiveness of this strategy depends on local community participation. The ornamental value of *C. panzhihuaensis* makes it vulnerable to illegal collection, a threat that can be best mitigated by involving residents as active stewards of conservation. We suggest developing co-management programs that provide local communities with tangible incentives, such as direct subsidies, alternative livelihood options, or ecotourism opportunities, to engage in monitoring, restoration, and protection activities. By combining scientific prioritization with community-based action, this framework not only enhances the representativeness of the existing protected area network but also ensures that conservation efforts remain socially feasible, economically sustainable, and ecologically effective.

### 3.4. Implications for Conservation Strategies of Rare Plants in the HDM

Unlike the broadly distributed tropical and subtropical species of *Cycas*, which are primarily shaped by climatic variables [49], mountain-endemic taxa such as *C. panzhihuaensis* exhibit distribution patterns that are more strongly driven by the synergistic effects of multiple environmental factors [33,36]. Steep terrain in mountainous regions generates pronounced local climatic gradients, which in turn amplify species’ sensitivity to microhabitat heterogeneity [50]. Therefore, conservation strategies should move beyond climate-centric modeling frameworks and incorporate topography, anthropogenic disturbances, soil conditions, and their interactions to improve the accuracy and ecological relevance of habitat suitability assessment.

Furthermore, it is important to recognize the differences between single-species and multi-species conservation in terms of goal setting and spatial scale. Narrow-ranged species such as *C. panzhihuaensis* rely heavily on specific niche conditions, including the hot and arid climate of dry–hot valleys and calcium-rich soil environments. These species typically exhibit limited distributions and high sensitivity to microhabitat variation. Therefore, conservation strategies should aim to precisely identify critical habitat features and account for the coupled effects of climate, topography, and soil at local scales. In contrast, multi-species conservation emphasizes the identification of common environmental constraints and key ecological processes, such as future climate change [41] and anthropogenic disturbances [51], to optimize protected area networks and improve landscape connectivity at broader regional scales. Accordingly, conservation efforts for rare plant species in the HDM should balance the habitat specificity of focal taxa with the ecological commonalities among multiple species, seeking coordination and complementarity between different conservation strategies to facilitate a shift from localized habitat protection toward integrated ecosystem restoration.

### 3.5. Limitations

It should be noted that our study has several limitations. Firstly, the assessment of human influences was based only on HFI, which does not capture fine-scale variation in specific pressures such as land-use changes, night-time lights, or road density. Furthermore, our analysis of soil factors focused on soil pH and exchangeable calcium due to their direct relevance to the growth and habitat preference of *C. panzhihuaensis*. However, other soil properties, including organic matter content, nitrogen, phosphorus, and soil texture, may also influence plant performance and distribution. Incorporating these additional variables in future studies could provide a more comprehensive understanding of edaphic constraints and improve the predictive power of habitat suitability models. Additionally, the limited number of survey points (*n* = 58) may affect the generalizability of our findings. Finally, while our study identified priority conservation areas using MaxEnt 3.4.4 and Zonation 5, we did not perform a quantitative comparison of protection scenarios, such as the potential gains from expanding the existing protected area network. Due to data and methodological constraints, the socio-economic feasibility and community participation aspects were not incorporated. Future research could integrate dynamic scenario modeling, socio-economic data, and local stakeholder engagement to evaluate the effectiveness and practical implementation of expanded conservation networks.

## 4. Materials and Methods

### 4.1. Study Area

The study area is situated along the eastern margin of the HDM, straddling the border between Sichuan and Yunnan Provinces (25°30′–27°50′ N, 100°15′–102°45′ E). It is embedded within the deeply dissected river valley system of the Jinsha and Yalong Rivers and represents a key ecotonal transition zone between the Qinghai–Tibet Plateau and the Yunnan–Guizhou Plateau. It is characterized by both the biodiversity richness of the HDM hotspot and the ecological vulnerability of dry–hot valley systems. Influenced by monsoonal circulation and foehn winds, the regional climate exhibits the typical characteristics of dry–hot valley monsoons, characterized by high temperatures, intense solar radiation, and pronounced seasonal drought. The terrain is dominated by low to mid-altitude mountains (700–3000 m), interspersed with secondary landforms such as river terraces and faulted basins. These features create a pronounced topographic gradient centered around the Jinsha and Yalong Rivers and their tributaries, contributing to the region’s distinct microclimatic and habitat heterogeneity [27,52].

Based on historical distribution records and recent field survey data, *C. panzhihuaensis* is primarily distributed at elevations between 700 and 2300 m. Accordingly, Elevation data at a 30 m resolution were obtained from the ASTER Global Digital Elevation Model (ASTER GDEM) [53], and were used to delineate the spatial extent of the study area. Elevation zones between 650 and 3050 m were extracted using the Mask tool in ArcGIS 10.8. The resulting study area covers a total of 68,321.96 km^2^, as shown in Figure 6.

### 4.2. Occurrence Records

Distribution data for *C. panzhihuaensis* were obtained through field surveys and supplemented with specimen records from the Global Biodiversity Information Facility (GBIF) [54] and the Chinese Virtual Herbarium (CVH) [55]. A total of 73 raw occurrence points were initially collected. To reduce spatial autocorrelation and sampling bias, records of cultivated individuals were excluded. A spatial filtering procedure was then applied using a 1 km buffer radius to remove duplicate points within proximity. After filtering, 58 unique occurrence records were retained for subsequent model construction.

### 4.3. Data Sources and Pre-Processing of Environmental Variables

In this study, 25 environmental variables were initially selected based on the ecological characteristics of *C. panzhihuaensis* (Table S1). These variables were grouped into four categories: climatic, topographic, soil, and anthropogenic factors.

To ensure spatial consistency, all environmental layers were resampled to a resolution of 1 km. To reduce multicollinearity among predictors, we calculated the Variance Inflation Factor (VIF) in R version 4.2.1. A stepwise regression procedure was employed to iteratively remove variables with VIF values exceeding 10 [56]. Ultimately, 11 variables were retained for subsequent analysis, as listed in Table 1.

#### 4.3.1. Climatic Variables

Nineteen bioclimatic variables were obtained from the WorldClim 2.1 database at a spatial resolution of 30 arc-seconds [57]. Current climate data represent average conditions for 1970–2000, while future climate data for 2021–2040 and 2041–2060 were derived from CMIP6 projections under three Shared Socioeconomic Pathways (SSP1-2.6, SSP2-4.5, and SSP5-8.5).

#### 4.3.2. Topographic Variables

Elevation, slope, and aspect were derived from the ASTER GDEM [53] using the Spatial Analyst module in ArcGIS 10.8.

#### 4.3.3. Soil Variables

Soil pH and exchangeable calcium were obtained from the gridded dataset of soil properties in China [58]. Soil pH affects nutrient availability and microbial activity [59], while exchangeable calcium reflects the calcium content derived from calcareous bedrock, which is the primary substrate for most *C. panzhihuaensis* populations [9].

#### 4.3.4. Anthropogenic Variable

Human disturbance was represented by the Human Footprint Index (HFI), which provides a cumulative measure of human pressure on terrestrial ecosystems [60]. The HFI integrates eight human pressure variables, including built environments, population density, nighttime lights, croplands, pastures, roads, railways, and navigable waterways. Each pressure layer was rescaled to a 0–10 scale according to its relative intensity, and the weighted layers were summed to obtain a composite index ranging from 0 to 50, with higher values indicating stronger anthropogenic disturbance. Furthermore, in order to visualize spatial patterns of human disturbance in our study area, we generated an HFI map (Figure 5), which highlights zones of high and low anthropogenic pressure relevant to *C. panzhihuaensis* distribution.

### 4.4. Habitat Suitability Modeling Using MaxEnt

The MaxEnt model is a widely used species distribution model that estimates potential habitat suitability based on known occurrence data and environmental variables, while requiring only presence data as input. It has been shown to perform robustly in ecological studies, particularly for rare and narrowly distributed species [18,19]. In this study, we used MaxEnt version 3.4.4 to predict suitable habitats, with the following parameter settings: 70% of the occurrence data were used for model training and 30% for testing. The number of iterations was set to 10,000, and the model was run 10 times independently. The cloglog output format was selected to better interpret probability values while reducing extrapolation risks [61].

Model performance was evaluated using the Receiver Operating Characteristic (ROC) curve, with the Area Under the ROC Curve (AUC) serving as the accuracy metric. According to the classification criteria proposed by [62], models with AUC > 0.9 are considered to have excellent predictive performance. Jackknife cross-validation was employed to quantify the contribution of individual variables and to identify key environmental drivers [25].

The MaxEnt output was imported into ArcGIS 10.8 for spatial processing and visualization. Suitable habitats were classified into four levels using the Jenks natural breaks method: unsuitable habitat (0–0.10), low suitability (0.10–0.32), medium suitability (0.32–0.62), and high suitability (0.62–1). To improve the accuracy of area calculations, spatial statistics were computed using the Albers equal-area conic projection.

### 4.5. Quantifying Driving Factors with Geodetector

The Geodetector model is a statistical tool designed to detect spatial stratified heterogeneity and to identify the driving forces behind observed spatial patterns [22]. Based on the optimal parameters, Geodetector identifies the spatial differentiation characteristics of driving factors by minimizing intra-group variance and maximizing inter-group differences through effective spatial stratification [63]. It is particularly suitable for ecological and geographical studies, as it quantifies the explanatory power of environmental variables and their interactions in shaping species distributions. In this study, we employed Geodetector to analyze the contribution of climatic, topographic, soil, and anthropogenic factors to the distribution of *C. panzhihuaensis*. The analysis was performed using the GD package in R version 4.2.1 which was developed by the original authors of the Geodetector model.

Two modules of Geodetector were applied in this study: the Factor Detector and the Interaction Detector. The Factor Detector quantifies the explanatory power of individual environmental factors by calculating the q-statistic through variance decomposition; a higher *q*-value indicates a stronger explanatory ability of the factor for spatial heterogeneity in species distribution [22,64]. The Interaction Detector assesses pairwise interactions between factors by comparing the *q*-value of combined factors with those of individual factors. This allows the identification of interaction types, such as nonlinear enhancement, bi-factor enhancement, independence, or weakening effects, and thus reveals potential synergistic or offsetting relationships among factors.

### 4.6. Identification of Spatial Conservation Prioritization

Based on the suitable habitats predicted by MaxEnt 3.4.4, the analysis of spatial conservation prioritization was carried out in Zonation 5 [65]. The Zonation conservation planning tool is a spatial prioritization software that ranks grid cells according to their biodiversity value while considering habitat quality and landscape connectivity. Compared with other tools, Zonation emphasizes both habitat quality and spatial connectivity through the core-area and edge-removal rules, which is critical for the conservation of fragmented and narrowly distributed species such as *C. panzhihuaensis* [66].

In response to the Post-2020 Global Biodiversity Framework established under the Convention on Biological Diversity, which includes a goal of conserving at least 30% of representative ecosystems by 2030 [67], we defined the top 30% of grid cells by priority ranking as priority conservation areas. For management feasibility, these areas were further stratified into three conservation levels: Level I (score ≥ 0.9), Level II (0.8 ≤ score < 0.9), and Level III (0.7 ≤ score < 0.8), providing a framework for implementing tiered protection and differentiated management strategies.

## 5. Conclusions

This study established an integrated analytical framework by combining species distribution modeling (MaxEnt 3.4.4), driving factor analysis (Geodetector), and spatial prioritization (Zonation 5) to systematically evaluate the distribution of suitable habitats, environmental drivers, and priority conservation areas for *C. panzhihuaensis*. The key findings are summarized as follows:

First, the suitable habitat of *C. panzhihuaensis* constituted only 18.36% of the study region and was predominantly distributed in a belt-like pattern along the dry–hot valleys of the Jinsha and Yalong Rivers. It exhibited marked spatial fragmentation, a narrow ecological niche, and high sensitivity to environmental disturbances, and is declining in area while shifting southward and southeastward under climate change.

Second, elevation emerged as the most critical single driving factor, while significant interactive effects among topography, climate, soil, and anthropogenic influences collectively shaped the spatial heterogeneity and distribution boundaries of suitable habitats. These results highlight that relying solely on single-factor models risks overlooking the complex coupling mechanisms that govern species distributions in montane systems.

Third, priority conservation areas were predominantly located in the mid- to high-elevation zones of the southern and western parts of the region. Notably, only 6.17% of these areas fall within existing protected areas, indicating a significant conservation shortfall and underscoring the urgent need to strengthen conservation efforts within biodiversity hotspots.

To provide actionable guidance for conservation, we recommend a phased approach: in the short term, implement targeted protection and monitoring in priority habitats to reduce anthropogenic disturbances such as illegal harvesting and habitat degradation; in the medium term, expand and optimize the protected area network to encompass underrepresented mid- to high-elevation habitats, and promote habitat restoration and reforestation to enhance connectivity among fragmented populations; in the long term, integrate species-specific conservation priorities into regional land-use planning and development policies, while encouraging local community participation and awareness programs to ensure sustainable coexistence between human activities and the conservation of *C. panzhihuaensis*.

From an academic perspective, our results provide new insights into how microhabitat heterogeneity and interactive effects among environmental drivers jointly shape the distribution of narrow endemic plants in montane ecosystems. This finding enriches the theoretical understanding of species–environment relationships in topographically complex landscapes and highlights the necessity of integrating multiple drivers rather than relying on single-factor models. From a conservation perspective, this study not only identifies distribution and priority conservation areas for *C. panzhihuaensis* but also provides a transferable framework for guiding conservation planning of other threatened taxa across the HDM and similar biodiversity hotspots worldwide.

## Figures and Tables

**Table 1:** Contribution and permutation importance of 11 environmental variables in MaxEnt.

Variables	Description and Unit	Percent Contribution (%)	Permutation Importance (%)
elev	Elevation (m)	43.1	48.7
bio14	Precipitation of Driest Month (mm)	16.9	11.7
HFI	Human Footprint Index	8.8	6.4
ex-Ca	Exchangeable calcium (me/100 g)	7.0	5.3
bio2	Mean Diurnal Range (°C)	6.8	10.7
bio18	Precipitation of Warmest Quarter (mm)	5.8	6.4
slope	Slope (°)	4.9	4.7
bio15	Precipitation Seasonality (mm)	3.2	0.4
pH	Soil pH value (H_2_O)	1.5	1.5
aspect	Aspect (°)	1.2	0.5
bio7	Temperature Annual Range (°C)	0.6	3.6

**Table 2:** Areas of suitable habitats for *Cycas panzhihuaensis* under current and future climate scenarios.

Period	Current	2021–2040			2041–2060		
		SSP126	SSP245	SSP585	SSP126	SSP245	SSP585
Low suitability (km^2^)	6935.82	3385.11	2693.4	3099.86	3878.37	1812.5	2097.20
Medium suitability (km^2^)	3549.03	1692.83	816.13	854.99	1887.5	395.75	363.30
High suitability (km^2^)	2056.92	752.19	202.89	165.73	749.88	68.02	48.60
Total suitable area (km^2^)	12,541.77	5830.13	3712.42	4120.58	6515.75	2276.27	2509.10

**Table 3:** Spatial coverage of priority conservation areas by existing protected areas.

Priority	County	Total Area (km^2^)	Area Covered by Existing Protected Areas (km^2^)	Coverage (%)
Level I	Renhe, Yanbian, Wuding, Huidong, Luquan, Dongchuan, Yongren, Yuanmou, Huaping, Xiqu, Dongqu, Huili, Qiaojia	1246.77	8.91	0.71
Level II	Renhe, Yanbian, Wuding, Huidong, Luquan, Dongchuan, Yongren, Yuanmou, Huaping, Xiqu, Dongqu, Butuo, Jinyang, Ningnan, Puge, Dechang, Qiaojia, Miyi, Yanyuan, Huili	1200.32	65.52	5.46
Level III	Renhe, Yanbian, Wuding, Huidong, Luquan, Dongchuan, Yongren, Huaping, Huili	1227.81	0.00	0.00

## Data Availability

The data generated during this study are available from the first author upon reasonable request. The data are not publicly available due to ongoing research.

## Supplementary Materials

The following supporting information can be downloaded at: https://www.mdpi.com/article/10.3390/plants14172797/s1, Figure S1: ROC curves of the MaxEnt model predicting the potential distribution of *C. panzhihuaensis*.; Figure S2: Spatial patterns of human disturbance in the study area. (a) HFI; (b) land-use types in 1990; (c) land-use types in 2020; Table S1: 25 Candidate Environmental Variables.
