# Supplementary material for: Habitat Suitability and Driving Factors of Cycas panzhihuaensis in the Hengduan Mountains
Source: Plants (Basel). 2025 Sep 6;14(17):2797. doi: 10.3390/plants14172797 (PMC12430310; doi:10.3390/plants14172797)
Supplement: Supplementary file 1 [file plants-14-02797-s001.zip › plants-3807844-supplementary.pdf]

Table S1. 25 Candidate Environmental Variables

Type	Variable Name	Description and unit
Climatic variables	bio1	Annual Mean Temperature (℃)
	bio2*	Mean Diurnal Range (℃)
	bio3	Isothermality
	bio4	Temperature Seasonality
	bio5	Max Temperature of Warmest Month (℃)
	bio6	Min Temperature of Coldest Month (℃)
	bio7*	Temperature Annual Range (℃)
	bio8	Mean Temperature of Wettest Quarter (℃)
	bio9	Mean Temperature of Driest Quarter (℃)
	bio10	Mean Temperature of Warmest Quarter (℃)
	bio11	Mean Temperature of Coldest Quarter (℃)
	bio12	Annual Precipitation (mm)
	bio13	Precipitation of Wettest Month (mm)
	bio14*	Precipitation of Driest Month (mm)
	bio15*	Precipitation Seasonality (mm)
	bio16	Precipitation of Wettest Quarter (mm)
	bio17	Precipitation of Driest Quarter (mm)
	bio18*	Precipitation of Warmest Quarter (mm)
	bio19	Precipitation of Coldest Quarter (mm)
Topographic variables	elev*	Elevation (m)
	aspect*	Aspect (°)
	slope*	Slope (°)
Edaphic variables	ex-Ca*	Exchangeable calcium (me/100g)
	pH*	Soil pH value (H^2O)
Anthropogenic variable	HFI*	Human Footprint Index

Note: * indicates the 11 variables selected for further modeling analysis

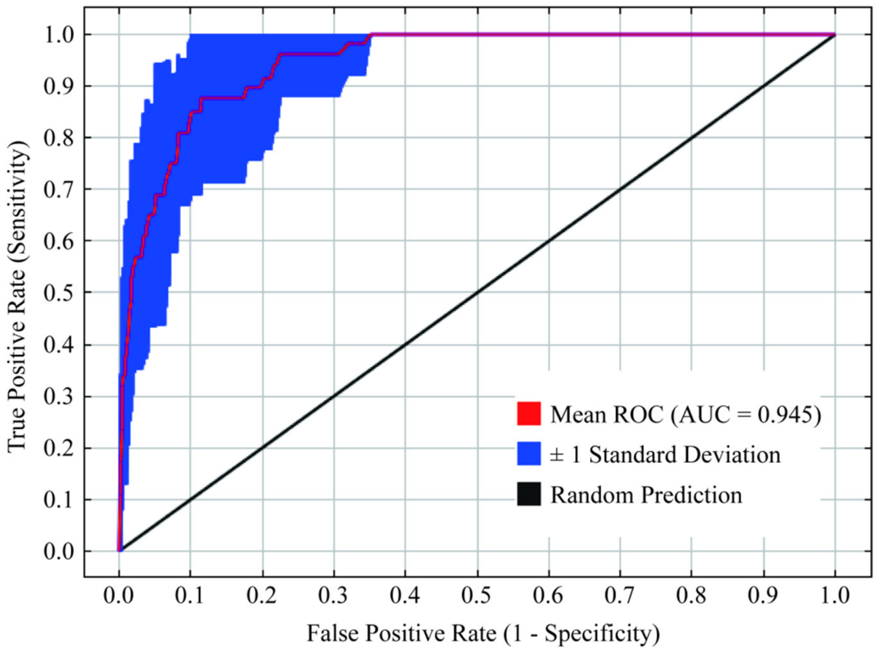

Figure S1. ROC curves of the MaxEnt model predicting the potential distribution of *C. panzhihuaensis*.

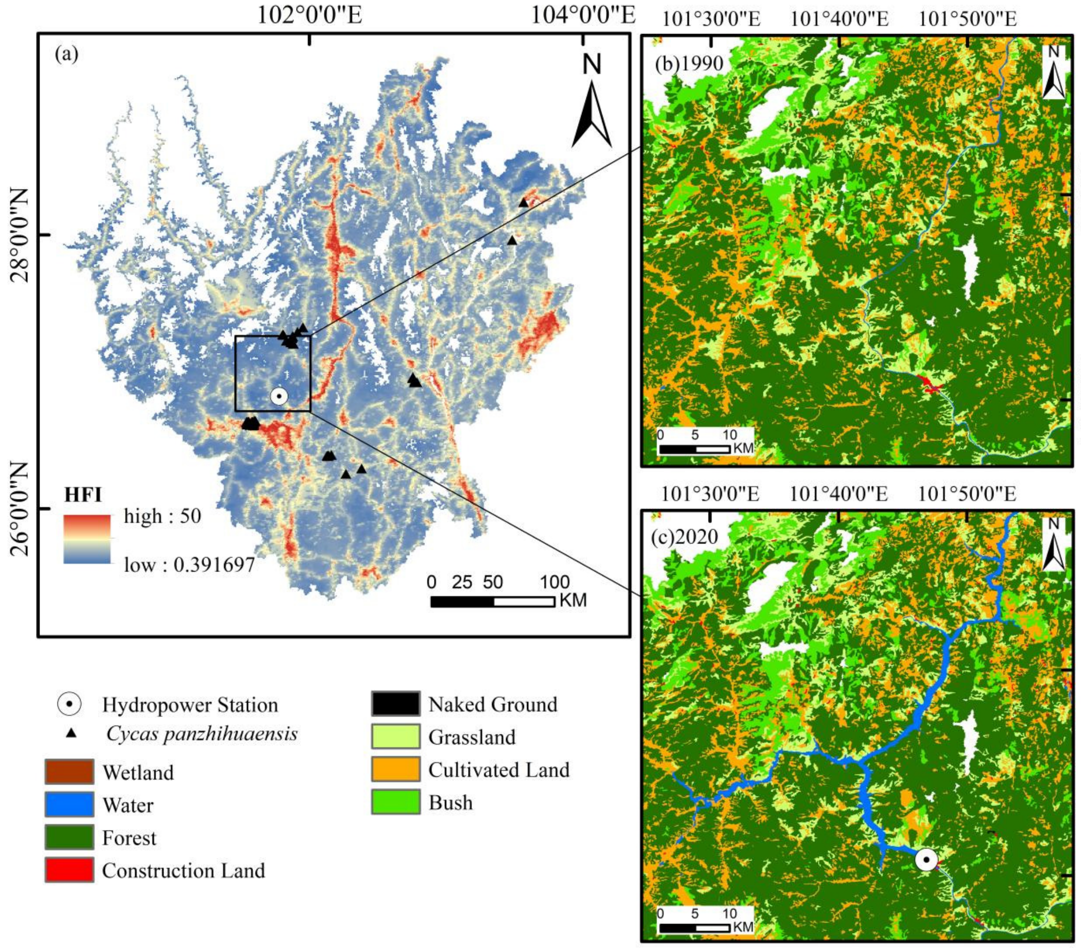

Figure S2. Spatial patterns of human disturbance in the study area. (a) HFI; (b) land-use types in 1990; (c) land-use types in 2020.
